# Supplementary material for: Single-cell profiling identifies IL1Bhi macrophages associated with inflammation in PD-1 inhibitor-induced inflammatory arthritis
Source: Nat Commun. 2024 Mar 7;15:2107. doi: 10.1038/s41467-024-46195-x (PMC10920757; doi:10.1038/s41467-024-46195-x)
Supplement: Supplementary file 1 — Supplementary Information [file 41467_2024_46195_MOESM1_ESM.pdf]

Supplementary Information

Single-cell profiling identifies *IL1B*^{hi} macrophages associated with inflammation in PD-1 inhibitor-induced inflammatory arthritis

Ziyue Zhou[1,2,3]†, Xiaoxiang Zhou[4]†, Xu Jiang[3,5]†, Bo Yang[6]†, Xin Lu[6], Yunyun Fei[1,2,3], Lidan Zhao[1,2,3], Hua Chen[1,2,3], Li Zhang[7], Xiaoyan Si[7], Naixin Liang[8], Yadong Wang[8], Dan Yang[1,2,3], Yezi Peng[1,2,3], Yiying Yang[1,2,3], Zhuoran Yao[9], Yangzhige He[3,5], Xunyao Wu[3], Wen Zhang[1,2,3], Min Wang[10], Huaxia Yang[1,2,3*], Xuan Zhang[10*]

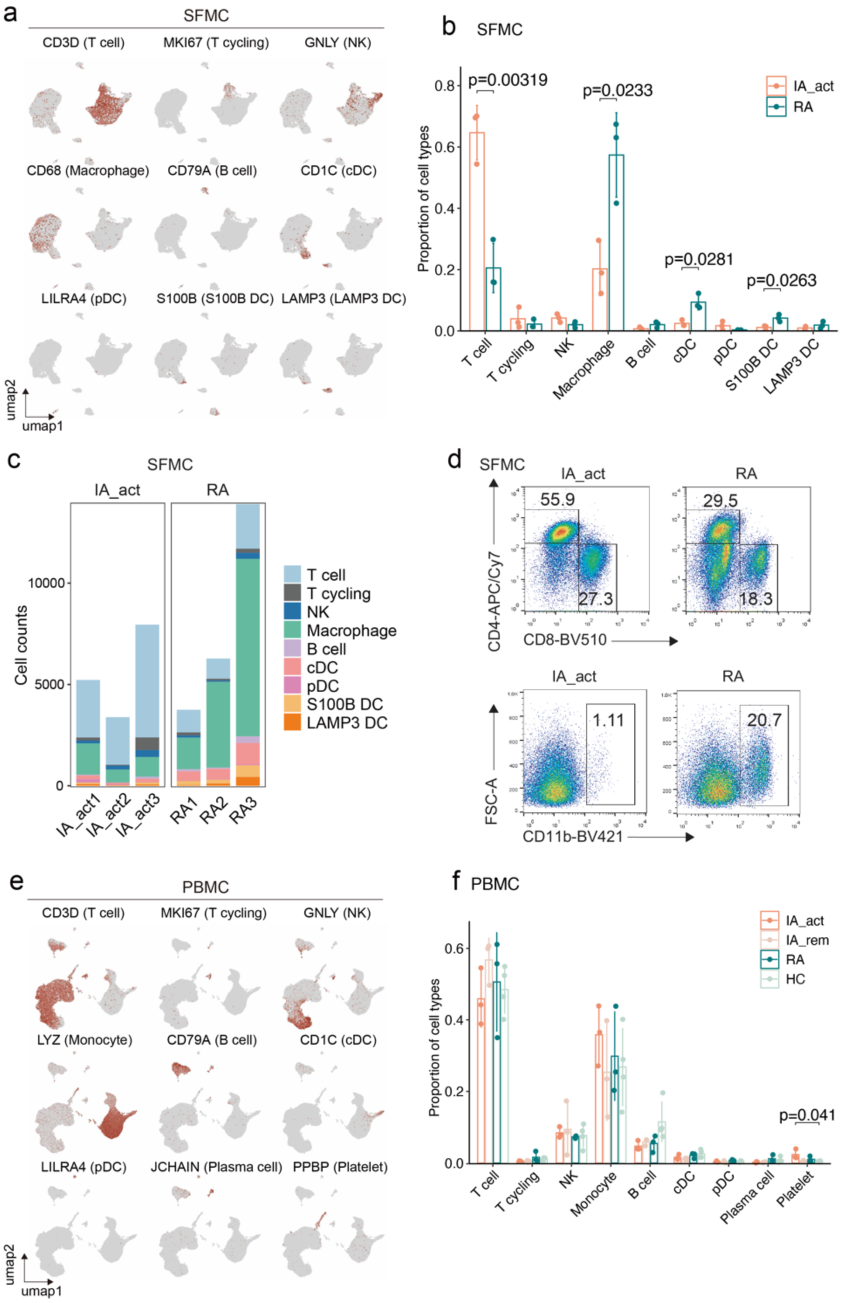

Supplementary Fig. 1. Canonical markers and cell frequencies of the major cell clusters in PD-1-IA patients and controls. **a.** UMAP visualization of the canonical markers of the major cell clusters in SFMCs. **b.** Quantification of the proportion (mean ± SD) of each major cell cluster in SFMCs between IA_act and RA with two-sided *t*-tests (n = 3 individuals per group). **c**. The cell counts of major cell types in SFMCs from all the individuals in the IA_act and RA group. **d.** Flow cytometry plots presenting T cells and macrophage subsets in SFMCs in IA_act and RA. **e.** UMAP visualization of the canonical markers of major cell clusters in PBMCs. **e.** Quantification of the proportion (mean ± SD) of each major cell cluster in SFMCs in each patient group (IA_act, IA_rem, RA, and HC, n = 3-4 individuals per group) with one-way ANOVA tests. Data are presented as mean (± SD). IA_act, active inflammatory arthritis. IA_rem, inflammatory arthritis in remission. RA, rheumatoid arthritis. HC, healthy control. T cycl., cycling T cells.

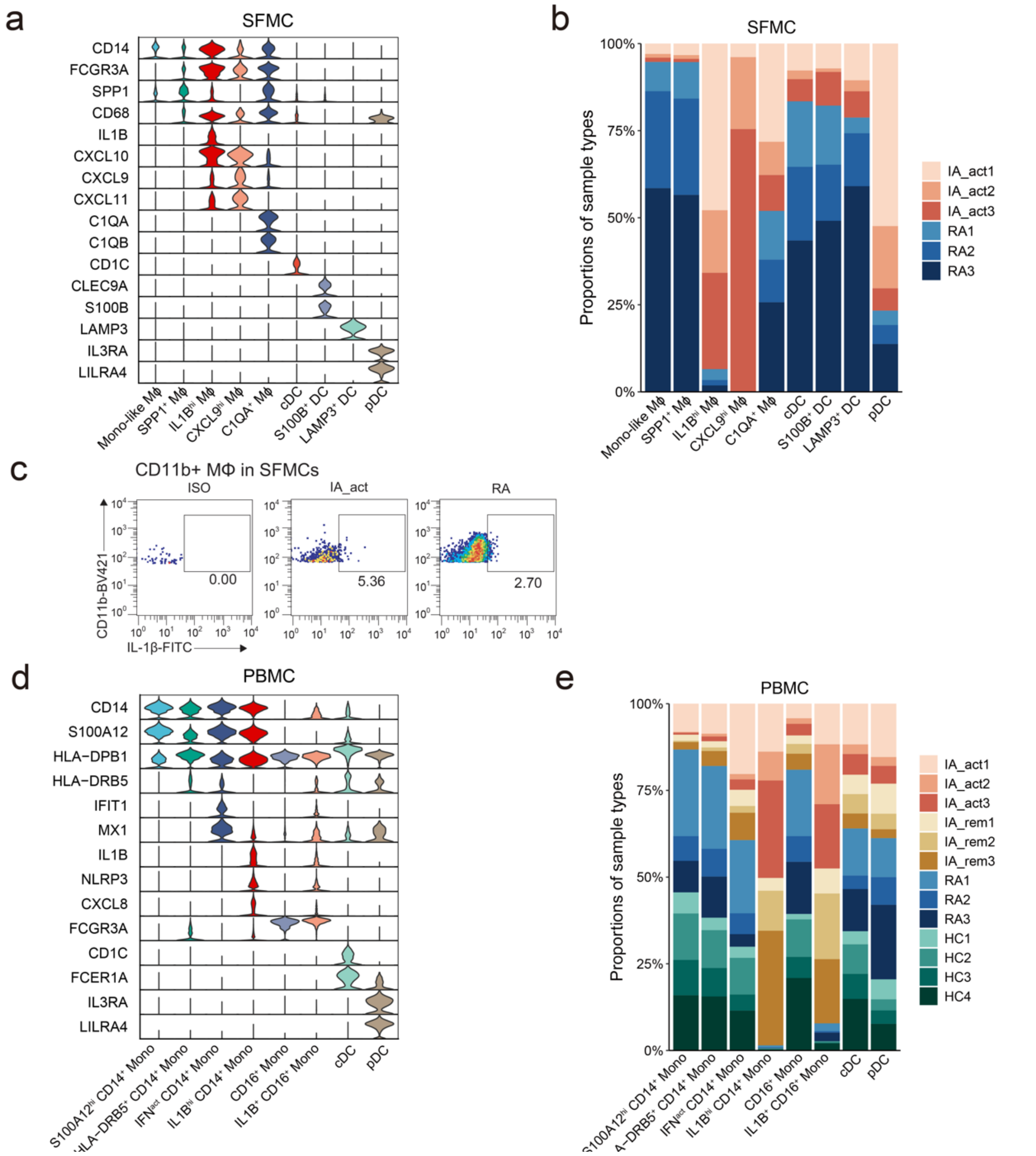

Supplementary Fig. 2. Marker genes and frequencies of the myeloid subclusters in SFMCs and PBMCs. **a.** Violin plots showing the marker genes of myeloid subclusters in SFMCs. **b.** Proportions of the sample sources within each myeloid subclusters in SFMCs, demonstrating that all the subclusters are composed of cells from multiple patient groups and thus are not affected by individual-specific batch effects. **c.** Quantification of IL1β^{+} macrophages in 1 IA_act sample and 1 RA sample (percentage of IL1β^{+} cells in CD11b^{+} cells) by flow cytometry. **d.** Violin plots showing the marker genes expression of the myeloid subclusters in PBMCs. **e.** Proportions of the sample sources within each myeloid subclusters in PBMCs, demonstrating that all subclusters are composed of cells from multiple patient groups and thus are not affected by individual-specific batch effects. ISO, isotype. IA_act, active inflammatory arthritis. IA_rem, inflammatory arthritis in remission. RA, rheumatoid arthritis. HC, healthy control.

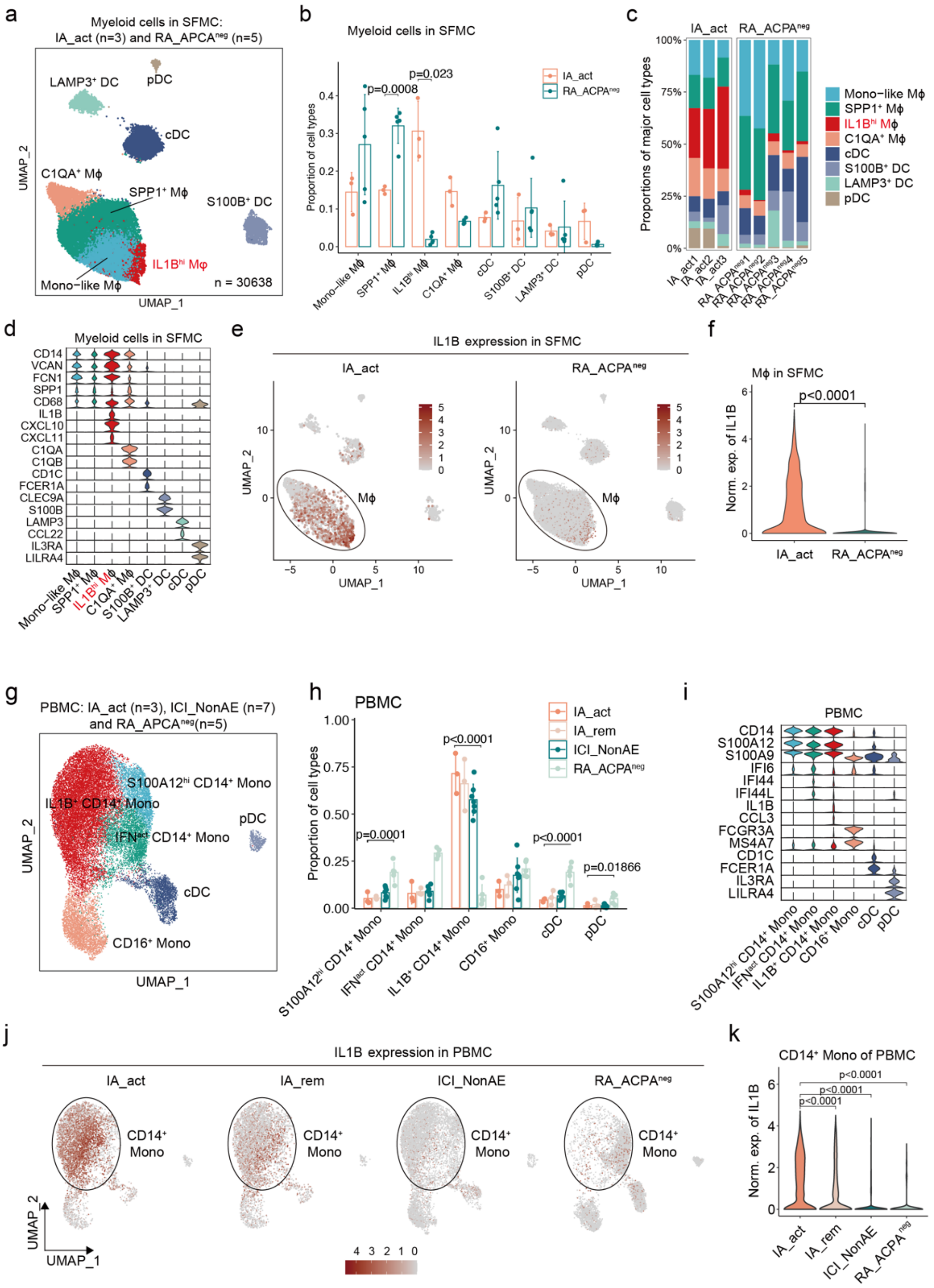

Supplementary Fig. 3. Myeloid subclusters in SFMCs and PBMCs of active IA patients compared with seronegative RA patients and ICI-treated patients without IA. **a.** Identification of 9 subclusters of myeloid cells across all SFMC samples. **b.** Quantification of the proportion (mean ± SD) of each myeloid cell subcluster in the SFMCs between IA_act and seronegative RA. **c.** The proportions of major cell types in SFMCs from all individuals in the IA_act and seronegative RA. **d.** Violin plots showing the marker genes expression of the myeloid subclusters in SFMCs. **e.** UMAP plot showing the *IL1B* gene expression of myeloid cells in PBMCs between IA_act and seronegative RA. **f.** Violin plot showing the *IL1B* gene expression of the macrophages in SFMCs between IA_act and seronegative RA. **g.** Identification of 8 subclusters of myeloid cells across all PBMC samples. **h.** Quantification of the proportion (mean ± SD) of each myeloid cell subcluster in the PBMCs

among the patient groups. **i.** Violin plots showing the marker genes expression of the myeloid subclusters in PBMCs. **j.** UMAP plots showing the *IL1B* gene expression of myeloid cells in PBMCs among the patient groups. **k.** Violin plot showing the *IL1B* gene expression of the $CD14^+$ monocytes in PBMCs among the patient groups. IA_act, active inflammatory arthritis. IA_rem, inflammatory arthritis in remission. ACPA, anti-citrullinated protein antibodies. RA, rheumatoid arthritis. HC, healthy control. MΦ, macrophages.

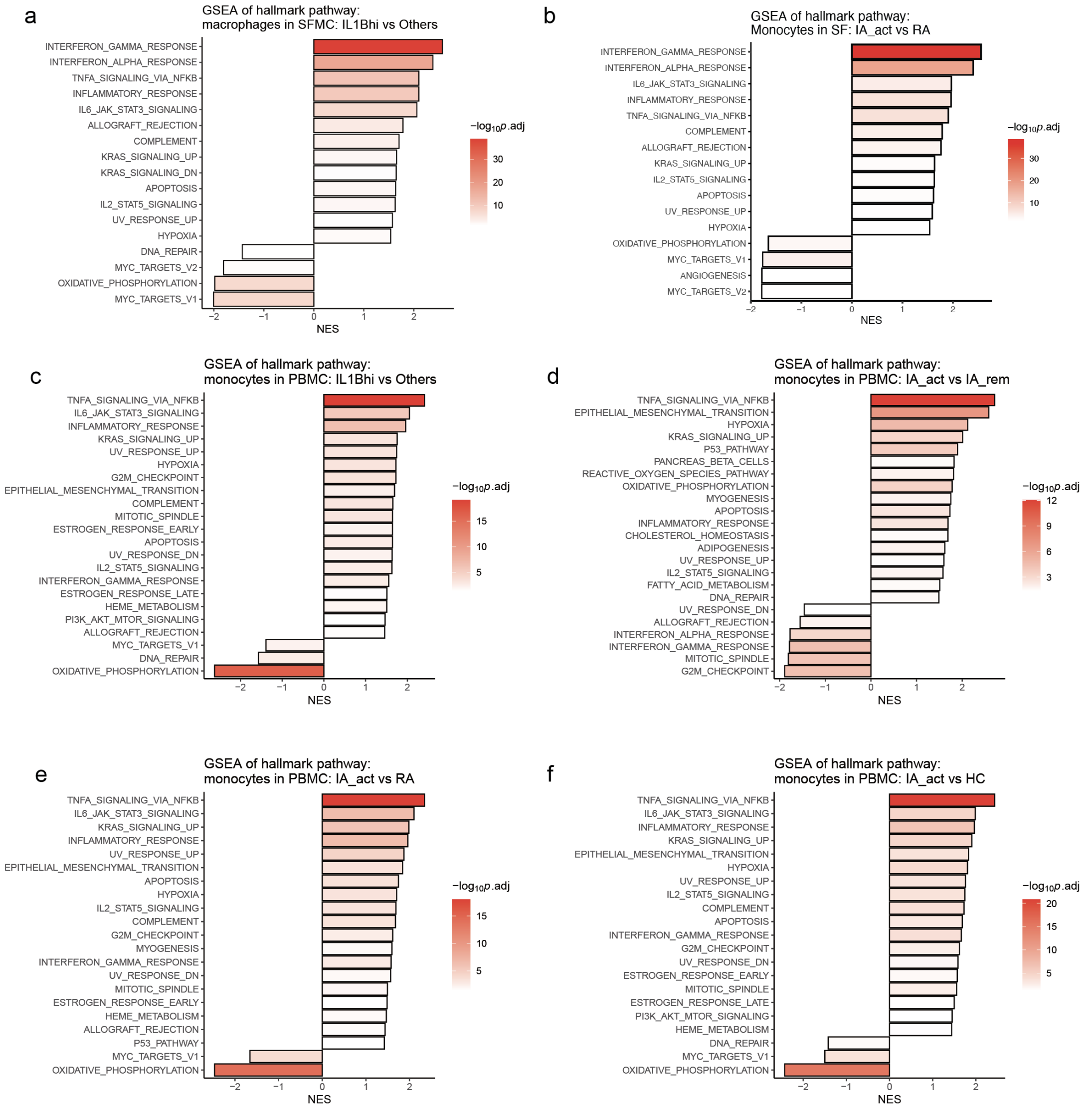

Supplementary Fig. 4. Upregulated Molecular Signatures Database (MsigDB) hallmark pathways of specific myeloid subclusters. a-b. Bar plot showing the significantly upregulated pathways of *IL1B*^{hi} macrophages compared with other macrophages (**a**) and macrophages in IA_act compared with RA (**b**) in SFMCs. **c.** Bar plot showing the significantly upregulated pathways of *IL1B*^{hi} monocytes compared with other monocyte subsets in PBMCs. **d-f.** Bar plot showing the significantly upregulated pathways of monocytes in PBMCs in IA_act compared with IA_rem (**d**), in IA_act compared with RA (**e**) and in IA_act compared with HC (**f**). IA_act, active inflammatory arthritis. IA_rem, inflammatory arthritis in remission. RA, rheumatoid arthritis. HC, healthy control. MΦ, macrophages.

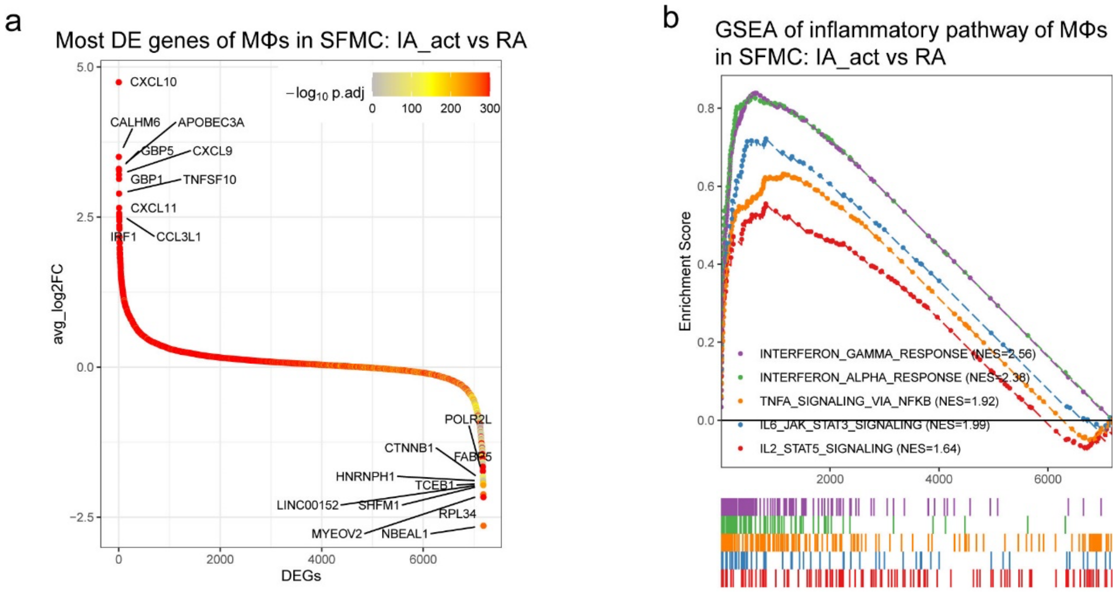

Supplementary Fig. 5. Differentially expressed genes of macrophages in SFMCs in active IA compared with RA. a. Differentially expressed genes between the macrophages in SFMCs in IA_act and RA. The top 10 and bottom 10 differentially expressed genes were labeled. **b.** Gene set enrichment analysis (GSEA) showing the upregulated the inflammatory pathways of macrophage/monocytes in SFMCs in IA_act, compared with RA. IA_act, active inflammatory arthritis. RA, rheumatoid arthritis. MΦ, macrophages.

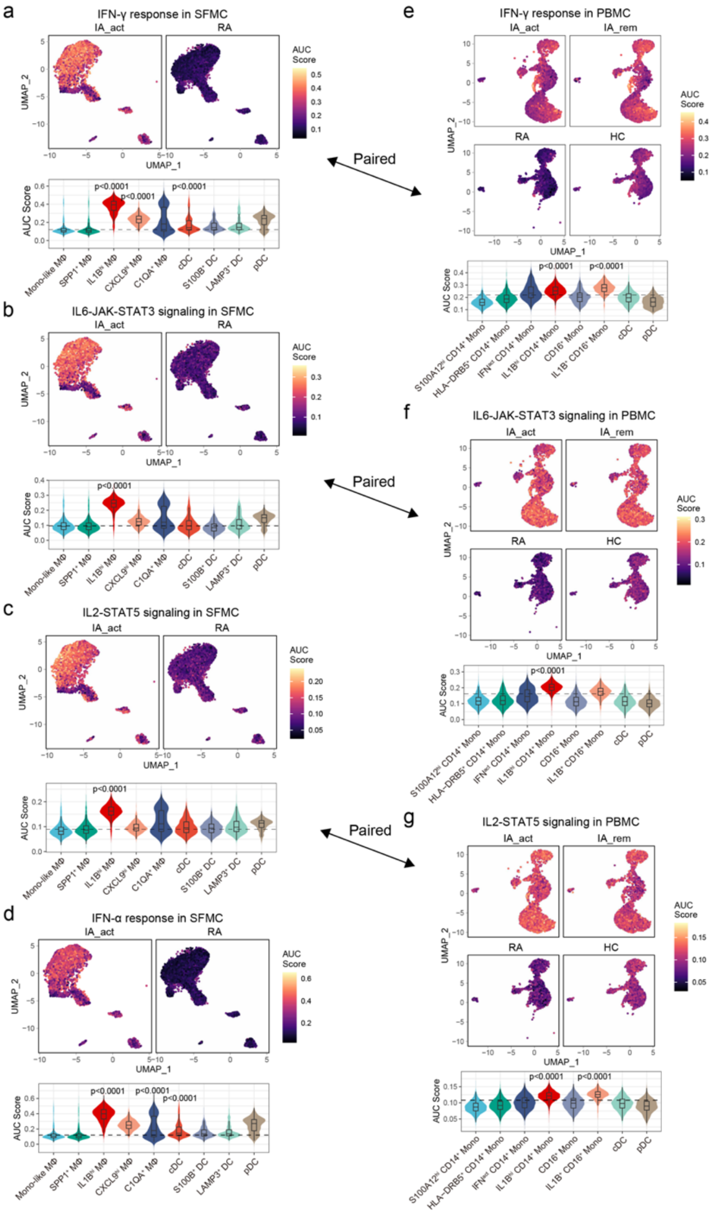

Supplementary Fig. 6. Signature scores of upregulated pathways across myeloid cells in SFMCs and PBMCs. a-d. UMAP (top) and violin and box plot (bottom) (median ± IQR) showing the signature score of IFNγ response pathway (**a**), IL6-JAK-STAT3 signaling pathway (**b**), IL2_STAT5 signaling pathway (**c**) and IFNα response pathway (**d**) among myeloid subclusters in SFMCs in IA_act and RA. **e-g.** UMAP (top) and violin and box plot (bottom) (median ± IQR) showing the signature score of IFNγ response (**e**), IL6-JAK-STAT3 signaling pathway (**f**) and IL2-STAT5 signaling pathway (**g**) pathway among myeloid subclusters in PBMCs in the patient groups (IA_act, IA_rem, RA and HC). The signature scores were calculated by the AUCell algorithm (see Methods). Two-sided Wilcoxon test comparing one subclusters and all other clusters are applied in the bottom panel of (**a-g**). The horizontal dashed lines in the violin plots denote the median expression of all the selected cell clusters, and only the clusters with expression significantly above the median are denoted. for all statistical tests in this figure. The double-sided arrows denoted the same pathway paired for SFMCs and PBMCs. IA_act, active inflammatory arthritis. IA_rem, inflammatory arthritis in remission. RA, rheumatoid arthritis. HC, healthy control. MΦ, macrophages.

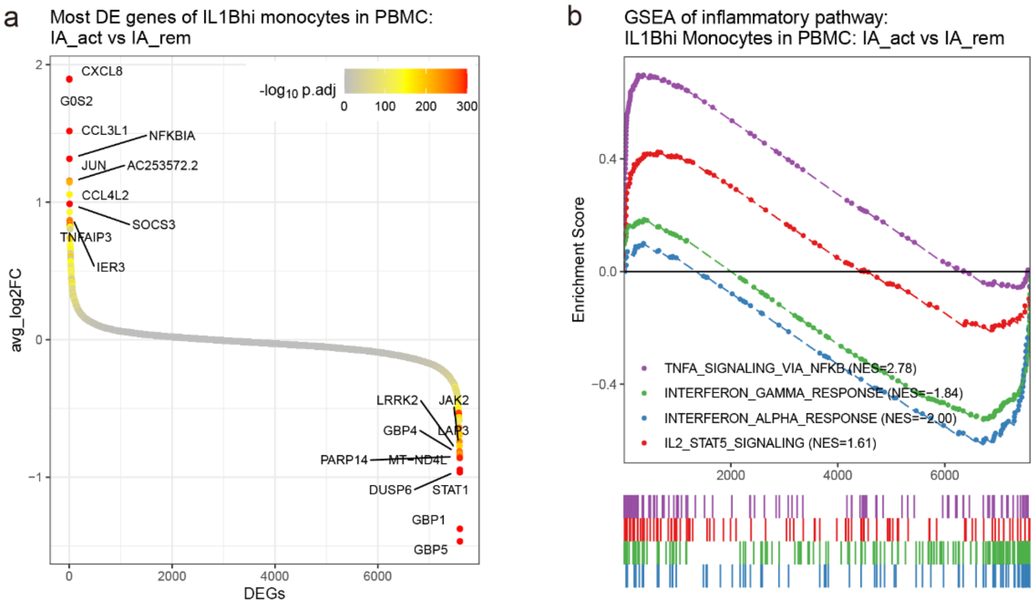

Supplementary Fig. 7. Differentially expressed genes and altered inflammatory pathways of the IL1B^{hi} monocytes in PBMCs in IA_act compared with IA_rem. a. DE genes of IL1B^{hi} monocyte subsets in PBMCs in IA_act compared with IA_rem. The top 10 and bottom 10 DE genes were labeled in the graph. **b.** GSEA showing the significantly altered inflammatory pathways of IL1B^{hi} monocytes in PBMCs in IA_act compared with IA_rem. DE genes, differentially expressed genes. GSEA, Gene set enrichment analysis. IA_act, active inflammatory arthritis. IA_rem, inflammatory arthritis in remission.

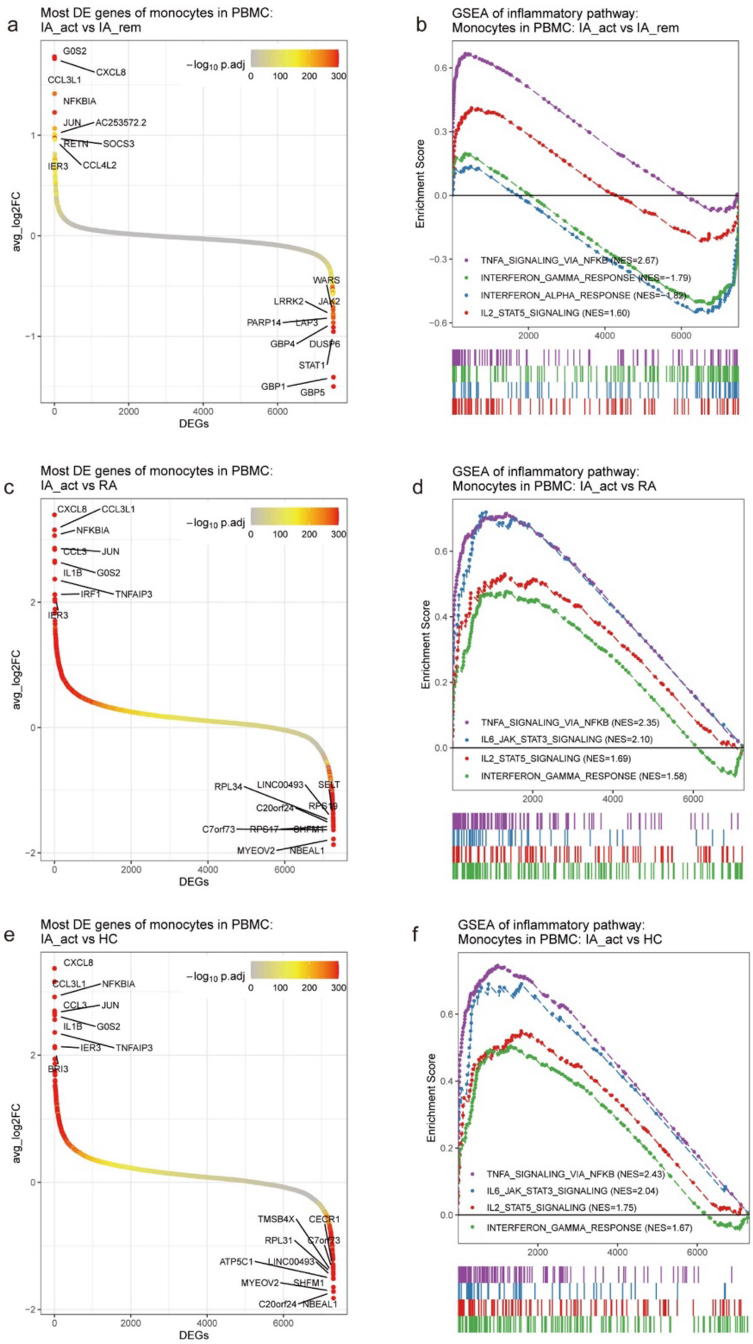

Supplementary Fig. 8. Differentially expressed genes and altered inflammatory pathways of monocytes in PBMCs in IA_act compared with controls. a. DE genes of monocytes in PBMCs between IA_act and IA_rem. **b.** GSEA showing the significantly altered inflammatory pathways of monocytes in PBMCs in IA_act compared with IA_rem. **c.** DE genes of monocytes in PBMCs between IA_act and RA. **d.** GSEA showing the significantly upregulated inflammatory pathways of monocytes in PBMCs in IA_act compared with RA. **e.** DE genes of monocytes in PBMCs between IA_act and HC. **f.** GSEA showing the significantly upregulated inflammatory pathways of monocytes in PBMCs in IA_act compared with HC. The top 10 and bottom 10 DE genes were labeled in (**a**), (**c**) and (**e**). DE genes, differentially expressed genes. GSEA, Gene set enrichment analysis. IA_act, active inflammatory arthritis. IA_rem, inflammatory arthritis in remission. RA, rheumatoid arthritis. HC, healthy control.

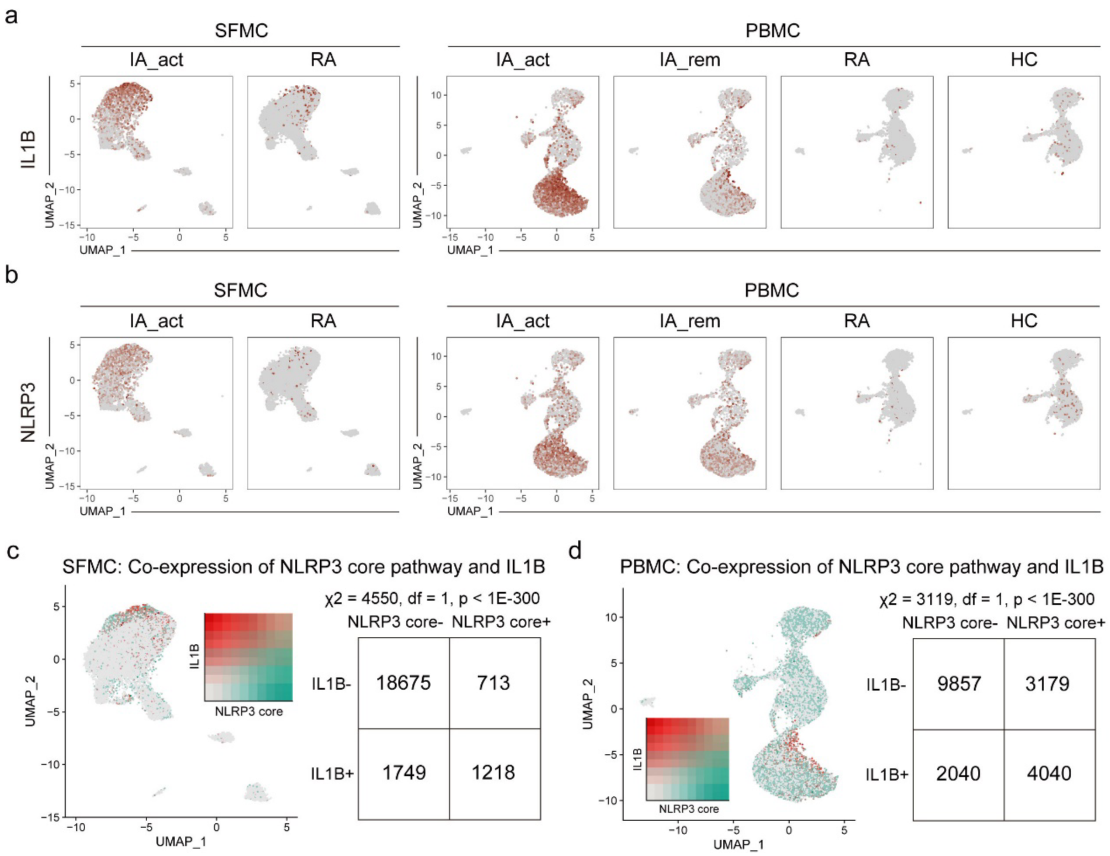

Supplementary Fig. 9. The association of *IL1B* gene expression with NLRP3 core pathway activity. a. UMAP plots of *IL1B* gene expression across myeloid cells in each patient group in SFMCs (left) and PBMCs (right). **b.** UMAP plots of *NLRP3* gene expression across the myeloid cells in SFMCs (left) and PBMCs (right) in each patient group. **c.** UMAP plot (left) showing the co-expression of NLRP3 core pathway activity and *IL1B* gene in myeloid cells in SFMCs. Chi-square test (right) examining the association of NLRP3 core pathway positive cells with *IL1B*$^+$ cells in myeloid cells in SFMCs. **d.** UMAP plot (left) showing the co-expression of NLRP3 core pathway activity and *IL1B* gene in myeloid cells in PBMCs. Chi-square test (right) examining the association of NLRP3 core pathway positive cells with *IL1B*$^+$ cells in myeloid cells in PBMCs. IA_act, active inflammatory arthritis. IA_rem, inflammatory arthritis in remission. RA, rheumatoid arthritis. HC, healthy control.

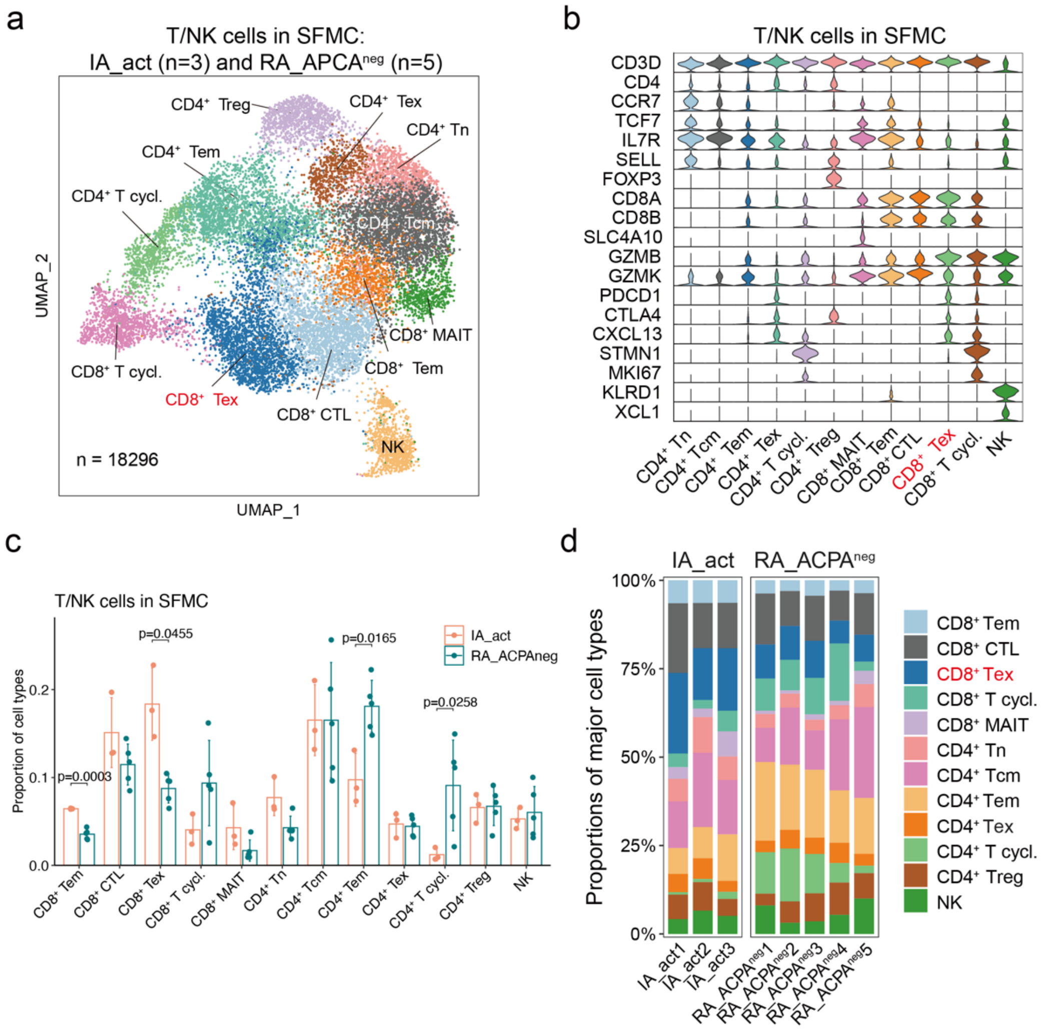

Supplementary Fig. 10. CD8$^+$ T-cell exhaustion in SFMCs of active IA compared with seronegative RA. **a.** Identification of 12 subclusters of T/NK cells across all SFMC samples (active IA and seronegative RA). **b.** Violin plots showing the expression of the marker genes of T/NK subclusters in SFMCs. **c.** Quantification of the proportion (mean ± SD) of each T/NK cell subcluster in the SFMCs between IA_act and seronegative RA. **d.** The proportions of major T/NK cell types in SFMCs from all the individuals in the patient groups (IA_act and seronegative RA). *p < 0.05, **p < 0.01, ***p < 0.001, ****p < 0.0001 for all statistical tests in this figure. IA_act, active inflammatory arthritis. RA, rheumatoid arthritis. T cycl., cycling T cells. NK, natural killer cells. ACPA, anti-citrullinated protein antibodies.

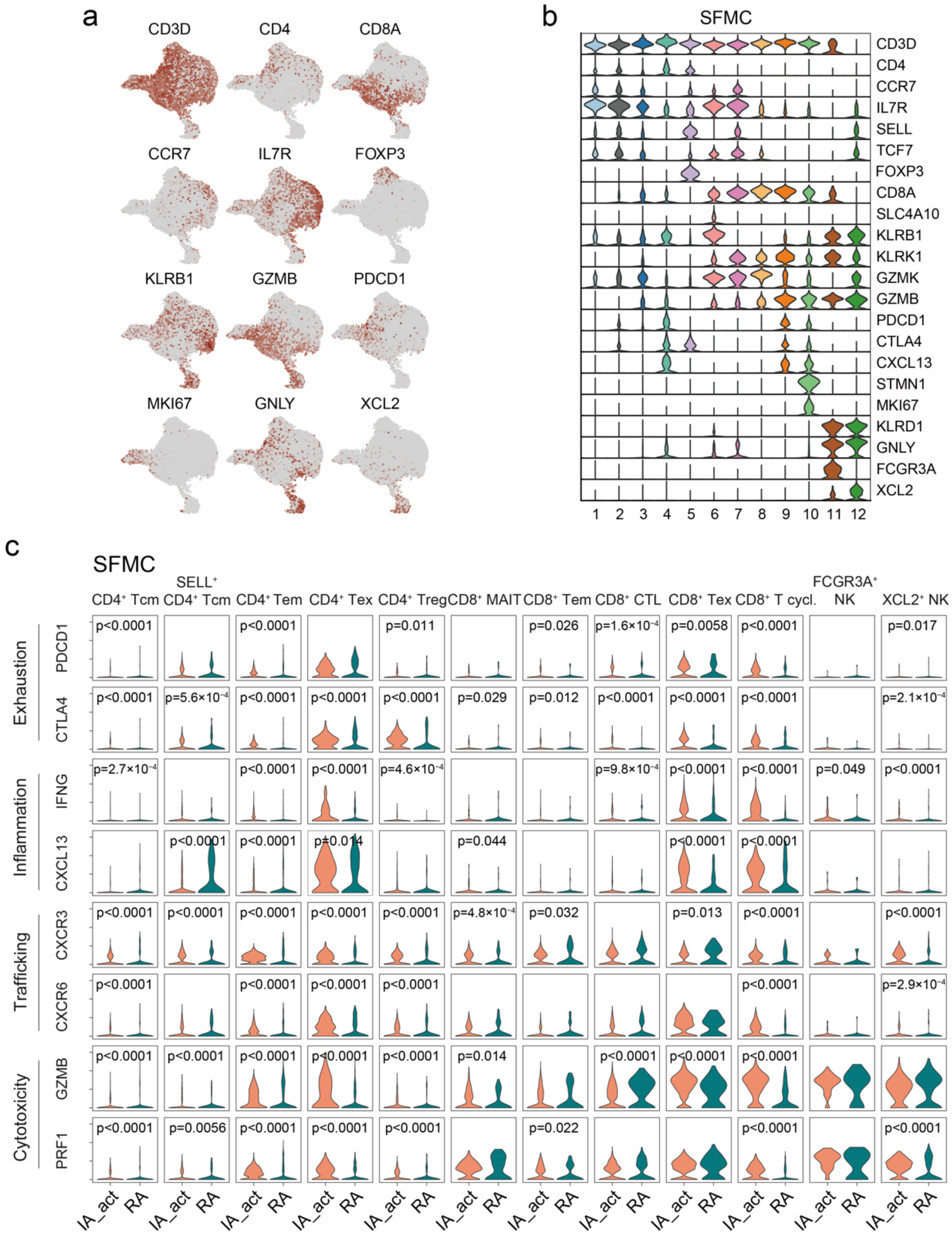

Supplementary Fig. 11. Marker genes of T/NK subclusters in SFMCs. **a.** UMAP visualization of the expression of the marker genes of T/NK subclusters in SFMCs. **b.** Violin plots showing the expression of the marker genes of T/NK subclusters in SFMCs. **c.** Violin plots showing the interested gene expression in all T/NK subclusters between the IA_act and RA. Two-sided Wilcoxon test comparing IA_act and RA. *p < 0.05, **p < 0.01, ***p < 0.001, ****p < 0.0001 for all statistical tests in this figure. IA_act, active inflammatory arthritis. RA, rheumatoid arthritis. T cycl., cycling T cells.

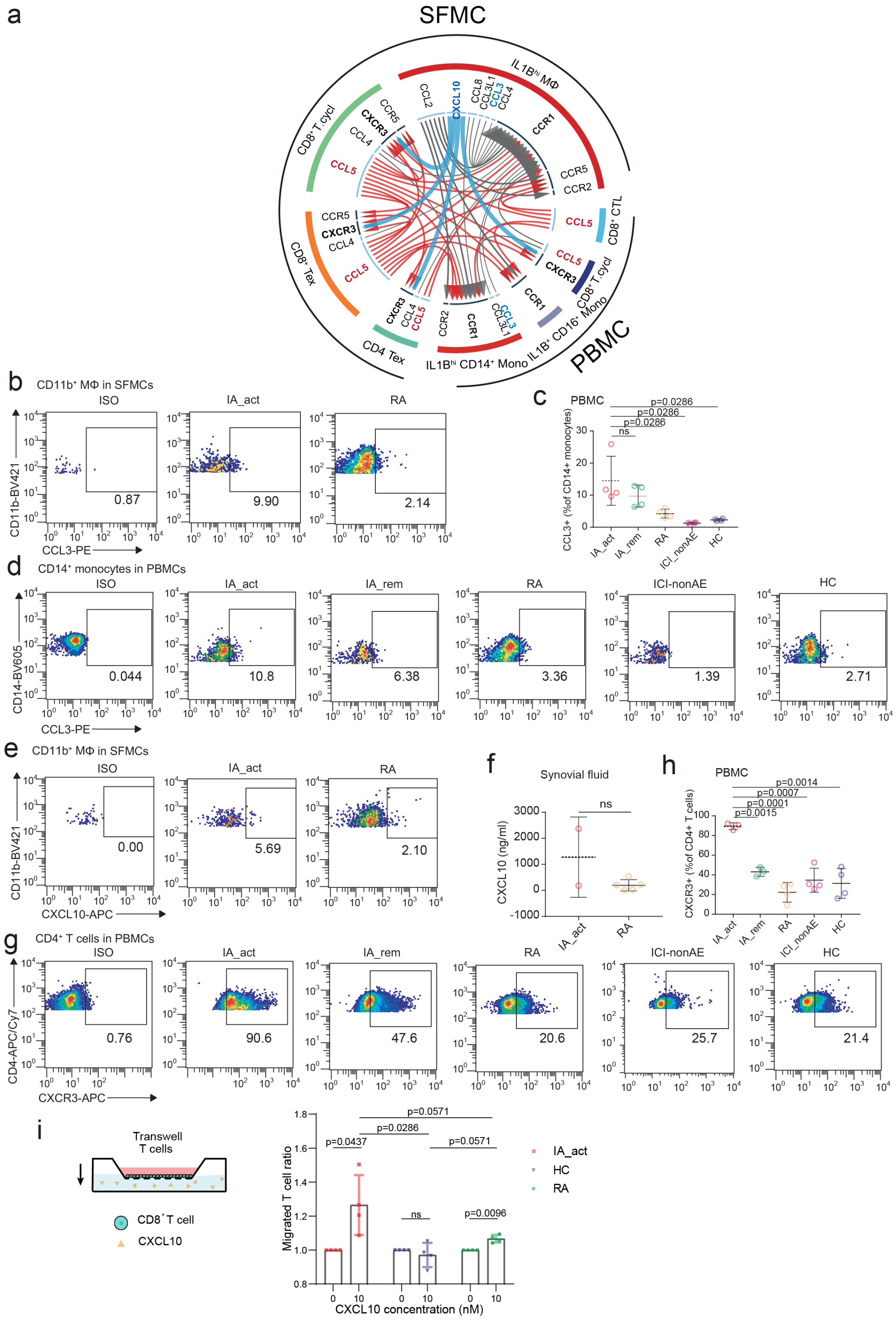

Supplementary Fig. 12. Crosstalk between *IL1B^hi* myeloid and T cells. a. Top 50 significantly enriched chemokine ligand-receptor pairs across *IL1B^hi* myeloid cells and T cells in PBMCs and SFMCs. **b.** Flow cytometry plot of CCL3$^+$ macrophages in SFMCs in IA_act and RA. **c.** Quantification of the proportions (mean ± SD) of CCL3$^+$ CD14$^+$ monocytes in PBMCs among patient groups (n=4 per group) by flow cytometry. The data show n=4 biological replicates over

three independent experiments. **d.** Representative flow cytometry plots for (**c**). **e.** Flow cytometry plot of CXCL10$^+$ macrophages (percentage of CXCL10$^+$ cells pre-gated on CD11b$^+$ macrophages) in SFMCs in IA_act and RA. **f.** Quantification of the synovial fluid CXCL10 protein concentration (mean ± SD) between IA_act and RA measured by ELISA. The data show n=2-5 biological replicates over two independent experiments. **h.** Quantification of the proportions (mean ± SD) of CXCR3$^+$ CD4$^+$ T cells in T cells in PBMCs among patient groups (n=3-4 per group) by flow cytometry. The data show n=3-4 biological replicates over three independent experiments. Paired two-sided *t* tests compared the IA_act group with the IA_rem group for (**c**) and (**h**). Wilcoxon tests compared the IA_act group with the RA, ICI-nonAE or HC group for (**c**), (**f**) and (**h**). **g.** Representative flow cytometry plots for (**h**). **i.** Migration of T cells under CXCL10 treatment by the chemotaxis assay. The migrated cell ratio (mean ± SD) was division of the migrated cell counts with CXCL10 treatment to those without CXCL10 treatment. The data show n=4 biological replicates over three independent experiments. Comparations were assessed by ratio paired t-test within each patient group or Wilcoxon tests among the patient groups. ns, non-significant. ISO, isotype. IA_act, active inflammatory arthritis. RA, rheumatoid arthritis. HC, healthy control. MΦ, macrophages. Mono, monocytes. T.cycl., cycling T cells.

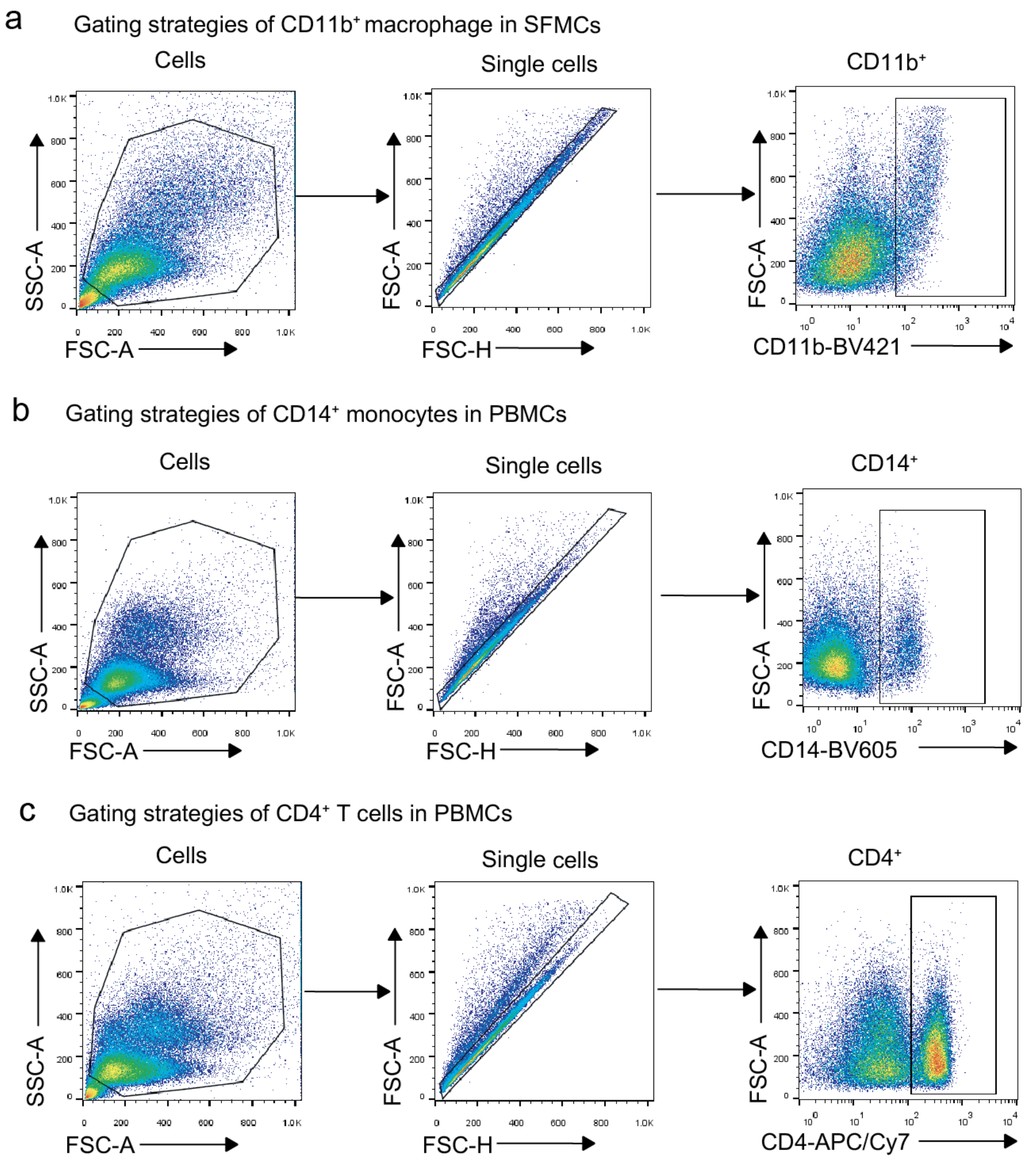

Supplementary Fig. 13. Gating strategies of monocytes and macrophages in flow cytometry. **a.** Gating strategies of $CD11b^+$ macrophage in SFMCs. **b.** Gating strategies of $CD14^+$ monocytes in PBMCs. **c.** Gating strategies of $CD4^+$ T cells in PBMCs.

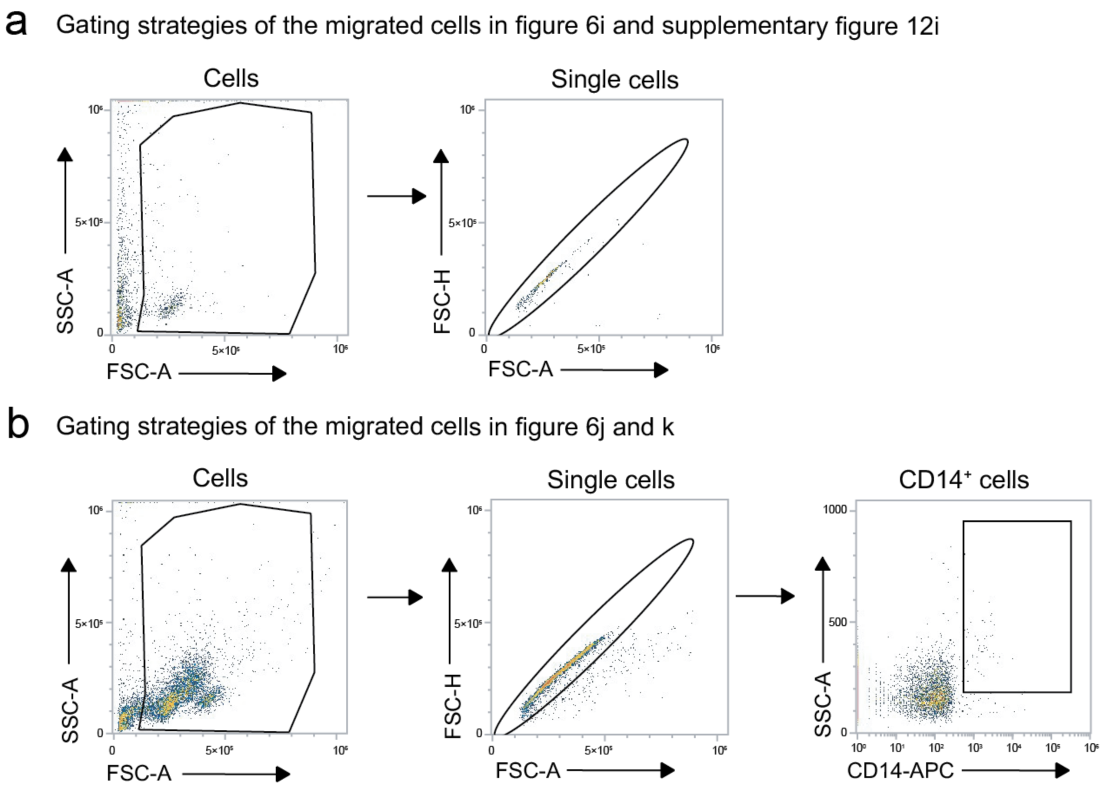

Supplementary Fig. 14. Gating strategies of the migrated cells for fluorescence activated cell sorting in the chemotaxis assay. a. Gating strategies of the single cells. **b.** Gating strategies of the migrated $CD14^+$ monocytes.

Supplementary Table 1. Demographics of enrolled patients with inflammatory arthritis and rheumatoid arthritis.

Patient	Age	Gender	Current Status (Until Nov. 2022)	ACPA	RF
IA_1	30	Female	alive	Negative	Negative
IA_2	59	Female	alive	Negative	Negative
IA_3	51	Male	alive	Negative	Negative
IA_4	65	Female	alive	Negative	Negative
IA_5	59	Female	alive	Negative	Negative
RA1	64	Female	alive	Positive	Positive
RA2	32	Female	alive	Positive	Positive
RA3	62	Female	alive	Positive	Positive
RA4	53	Female	alive	Positive	Positive
RA5	58	Male	alive	Positive	Positive
RA6	73	Female	alive	Positive	Positive
RA7	47	Female	alive	Positive	Negative
RA8	39	Female	alive	Positive	Positive

Abbreviations: ICI = immune checkpoint inhibitor; ACPA = Anticitrullinated-peptide antibodies; RF = rheumatoid factor; IA = inflammatory arthritis; RA = rheumatoid arthritis.

Supplementary Table 2. Baseline clinical characteristics of enrolled patients with inflammatory arthritis.

Patient	Malignancy	ICIs	IA onset* (days)	Treatment cycles†	CTCAE grade‡	Involved joints	Laboratory findings				Treatment of IA	Remission
							WBC ($\times10^9$/L)	LY ($\times10^9$/L)	ESR (mm/h)	hsCRP (mg/dL)		
IA_1	Esophageal squamous cancer	Pembrolizumab	30	4	3	Wrists, MCPs, PIPs, knees	7.97	1.65	96	21.12	TNF inhibitor, local GCs, NSAIDs	Yes
IA_2	Gallbladder mucinous adenocarcinoma	Camrelizumab	70	3	2	Knees	4	0.9	48	17.66	Local GCs, MTX, NSAIDs	Yes
IA_3	Esophageal squamous cancer	Camrelizumab	97	2	2	Knees	7.09	1.35	53	9.21	Local GCs, MTX, NSAIDs	Yes
IA_4	Small cell lung cancer	Pembrolizumab	387	29	3	MCPs, PIPs, knees	6.93	1.25	10	5.37	Local GCs, MTX, TII	Yes
IA_5	Small cell cervical cancer	Camrelizumab	151	9	2	MCPs, PIPs, hips, shoulders	6.6	1.56	85	11.43	Local GCs, MTX, NSAIDs	Yes

* IA onset was defined as the time interval between the first dose of ICI administration to IA onset.

† Treatment cycles were defined as the number of ICI cycles by the time of IA onset.

‡ CTCAE grade according to Common terminology criteria for adverse events.

Abbreviations: IA = inflammatory arthritis; ICI= immune checkpoint inhibitor; CTCAE = Common terminology criteria for adverse events; MCP = metacarpophalangeal joint; PIP =proximal interphalangeal joint; WBC = white blood cells; LY = lymphocyte; ESR = erythrocyte sedimentation rate; hsCRP = high-sensitivity C-reactive protein; TNF = tumor necrosis factor; NSAIDs = non-steroidal anti-inflammatory drugs; GC = glucocorticoid; MTX = methotrexate; TII = Tripterygium glycosides.
