# Supplementary material for: Single-cell profiling identifies IL1Bhi macrophages associated with inflammation in PD-1 inhibitor-induced inflammatory arthritis
Source: Nat Commun. 2024 Mar 7;15:2107. doi: 10.1038/s41467-024-46195-x (PMC10920757; doi:10.1038/s41467-024-46195-x)
Supplement: Supplementary file 3 — Reporting Summary [file 41467_2024_46195_MOESM3_ESM.pdf]

nature portfolio

Corresponding author(s): Huaxia Yang and Xuan Zhang

Last updated by author(s): Jan 15, 2024

Reporting Summary

Nature Portfolio wishes to improve the reproducibility of the work that we publish. This form provides structure for consistency and transparency in reporting. For further information on Nature Portfolio policies, see our Editorial Policies and the Editorial Policy Checklist.

Statistics

For all statistical analyses, confirm that the following items are present in the figure legend, table legend, main text, or Methods section.

n/a	Confirmed	
☐	☒	The exact sample size (*n*) for each experimental group/condition, given as a discrete number and unit of measurement
☐	☒	A statement on whether measurements were taken from distinct samples or whether the same sample was measured repeatedly
☐	☒	The statistical test(s) used AND whether they are one- or two-sided<br>*Only common tests should be described solely by name; describe more complex techniques in the Methods section.*
☒	☐	A description of all covariates tested
☒	☐	A description of any assumptions or corrections, such as tests of normality and adjustment for multiple comparisons
☐	☒	A full description of the statistical parameters including central tendency (e.g. means) or other basic estimates (e.g. regression coefficient) AND variation (e.g. standard deviation) or associated estimates of uncertainty (e.g. confidence intervals)
☐	☒	For null hypothesis testing, the test statistic (e.g. *F*, *t*, *r*) with confidence intervals, effect sizes, degrees of freedom and *P* value noted<br>*Give P values as exact values whenever suitable.*
☒	☐	For Bayesian analysis, information on the choice of priors and Markov chain Monte Carlo settings
☒	☐	For hierarchical and complex designs, identification of the appropriate level for tests and full reporting of outcomes
☒	☐	Estimates of effect sizes (e.g. Cohen's *d*, Pearson's *r*), indicating how they were calculated

Our web collection on statistics for biologists contains articles on many of the points above.

Software and code

Policy information about availability of computer code

Data collection	Single-cell RNA sequencing were performed on the Illumina NovaSeq6000 platform. FACSCelestaTM (BD Biosciences) for flow cytometry and FACS. Biotek SynergyNeo2 for ELISA.
Data analysis	Single-cell RNA-seq analysis, statistical analysis and graphing were performed using Cell ranger v6.1.2, FlowJo v10, GraphPad PRISM v8,Adobe Illustrator 2023 (Adobe), R software v4.1.2 and the following R packages: Seurat v4.0.5, Harmony v0.1.1, AUCell v3.17, ClusterProfiler v4.2.0, Monocle 3 v1.0.0, iTalk v0.1.0 and ggplot2 v3.3.5. No new algorithms were developed for this manuscript. All code generated for single-cell RNA-seq data analysis is available from the corresponding authors upon reasonable request.

For manuscripts utilizing custom algorithms or software that are central to the research but not yet described in published literature, software must be made available to editors and reviewers. We strongly encourage code deposition in a community repository (e.g. GitHub). See the Nature Portfolio guidelines for submitting code & software for further information.

Data

Policy information about availability of data

All manuscripts must include a data availability statement. This statement should provide the following information, where applicable:

- Accession codes, unique identifiers, or web links for publicly available datasets
- A description of any restrictions on data availability
- For clinical datasets or third party data, please ensure that the statement adheres to our policy

Sequence data generated in this work have been deposited in the Genome Sequence Archive in BIG Data Center, Beijing Institute of Genomics (BIG), Chinese

Academy of Sciences, under the accession code HRA004500 (https://ngdc.cncb.ac.cn/gsa-human/browse/HRA004500). Published single-cell expression datasets analyzed in this work are available from Genome Sequence Archive in BIG Data Center, Beijing Institute of Genomics (BIG), Chinese Academy of Sciences, under the accession code HRA000155 (https://ngdc.cncb.ac.cn/gsa-human/browse/HRA000155). Source data are provided with this paper.

Research involving human participants, their data, or biological material

Policy information about studies with human participants or human data. See also policy information about sex, gender (identity/presentation), and sexual orientation and race, ethnicity and racism.

Reporting on sex and gender	The detailed information on the sex of participants is provided in Supplementary Table 1 and 2. In summary, 4 female IA patients, 1 male IA patients, 7 female RA patients and 1 male RA patient participated in this study.
Reporting on race, ethnicity, or other socially relevant groupings	All the recruited patients are asians. No socially relevant categorization variables was used in this study.
Population characteristics	See Above.
Recruitment	We prospectively recruited adult patients with new-onset IA induced by PD-1 inhibitors (PD-1-IA). Active PD-1-IA (IA_act) was defined as: 1) presence of joint inflammation diagnosed by a rheumatologist based on the comprehensive assessment of the history, physical examination, inflammatory markers and imaging findings; 2) joint inflammation developed after anti-PD-1 administration. IA patients were excluded if they had pre-existing autoimmune disease.<br>Patients who fulfilled the 2010 American College of Rheumatology/European League Against Rheumatism (ACR/EULAR) classification criteria for RA were also recruited and further classified into seropositive or seronegative RA depending on the presence or absence of anticitrullinated-peptide antibodies (ACPA) and rheumatoid factor (RF).<br>Additionally, healthy donors with matched age and sex were recruited as control.
Ethics oversight	This study was performed following the ethical guidelines of the Declaration of Helsinki and was approved by the Peking Union Medical College Hospital Ethics Committee (no. JS-1940). Informed consent was obtained from all the participants before sample collection and clinical information.

Note that full information on the approval of the study protocol must also be provided in the manuscript.

Field-specific reporting

Please select the one below that is the best fit for your research. If you are not sure, read the appropriate sections before making your selection.

[x] Life sciences [ ] Behavioural & social sciences [ ] Ecological, evolutionary & environmental sciences

For a reference copy of the document with all sections, see nature.com/documents/nr-reporting-summary-flat.pdf

Life sciences study design

All studies must disclose on these points even when the disclosure is negative.

Sample size	No standard methods were used to predetermine sample size. We recruited 5 patients who were diagnosed with newly developed inflammatory arthritis and 8 patients who fulfilled the 2010 ACR/EULAR Rheumatoid Arthritis classification criteria. The sample sizes were sufficient to provide stable single cell clustering results and to perform statistical analysis.
Data exclusions	For scRNA-seq, doublets detected by Scrublet and doublet clusters expressing markers of more than two major cell lineages were removed from analysis. In addition, low quality cells meeting the following criteria were also removed: 1) number of genes detected per cell less than 200 or more than 5000; 2) UMIs detected per cell over 200; 3) counts of mitochondrial genes constituting over 12% of all gene counts; and 4) counts of red blood cell genes constituting over 0.3% of all gene counts.
Replication	We have repeated each experiment at least three time to ensure consistent results. All repeats performed showed similar trends.
Randomization	Randomization was not included in the study, as this study did not involve clinical trials/clinical trial associated data.
Blinding	Blinding of participants was not relevant to our study, for the main group of the study population was patients diagnosed with arthritis. For scRNA-seq, flow cytometry, ELISA and transwell experiments, the experimenters were blinded to the sample information.

Reporting for specific materials, systems and methods

We require information from authors about some types of materials, experimental systems and methods used in many studies. Here, indicate whether each material, system or method listed is relevant to your study. If you are not sure if a list item applies to your research, read the appropriate section before selecting a response.

Materials & experimental systems

n/a	Involved in the study
	[x] Antibodies
[x]	Eukaryotic cell lines
[x]	Palaeontology and archaeology
[x]	Animals and other organisms
	[x] Clinical data
[x]	Dual use research of concern
[x]	Plants

Methods

n/a	Involved in the study
[x]	ChIP-seq
	[x] Flow cytometry
[x]	MRI-based neuroimaging

Antibodies

Antibodies used	Antibodies were applied for flow cytometry and FACS, listed as following: anti-CD14-BV605 (#367126, clone 63D3, 1/20 dilution, BioLegend), anti-CD11b-BV421 (#301323, clone ICRF44, 1/20 dilution, BioLegend), anti-CD4-APC/Cy7 (#344615, clone SK3, BioLegend), anti-CD8-BV510 (#344731, clone SK1, 1/20 dilution, BioLegend), anti-IL-1β-FITC (#508206, clone JK1B-1, 1/20 dilution, BioLegend), anti-CCL3-PE (#12-9706-42, clone CR3M, 1/20 dilution, Thermo Fisher), anti-CCR1-APC/Cy7 (#362917, clone 5F10B29, 1/20 dilution, BioLegend), anti-CXCL10-APC (#519505, clone J034D6, 1/20 dilution, BioLegend), and anti-CXCR3-APC (#353707, clone G025H7, 1/20 dilution, BioLegend).
Validation	All antibodies were obtained commercially validated by the respective company. All antibodies had validation statement provided on the website of the manufacturer. Related technical data sheets can be obtained from the manufacturer's website using the catalog number provided above.

Clinical data

Policy information about clinical studies

All manuscripts should comply with the ICMJEguidelines for publication of clinical research and a completedCONSORT checklist must be included with all submissions.

Clinical trial registration	This is not a clinical trial.
Study protocol	Study design and recruitment of participants can be found in Methods section. Briefly, we prospectively recruited patients with newly developed inflammatory arthritis after immune checkpoint inhibitor administration and rheumatoid arthritis. Fresh synovial fluid samples were obtained from patients with active IA or active RA through arthrocentesis. Peripheral blood samples were collected from the patients through venepuncture. Mononuclear cells were isolated from synovial fluid and peripheral blood samples and were further used for sequencing and flow cytometry. Supernatant of synovial fluid and serum were used for ELISA tests.
Data collection	Clinical information were collected from medical records.
Outcomes	Not applicable.

Flow Cytometry

Plots

Confirm that:

- [x] The axis labels state the marker and fluorochrome used (e.g. CD4-FITC).
- [x] The axis scales are clearly visible. Include numbers along axes only for bottom left plot of group (a 'group' is an analysis of identical markers).
- [x] All plots are contour plots with outliers or pseudocolor plots.
- [x] A numerical value for number of cells or percentage (with statistics) is provided.

Methodology

Sample preparation	SFMCs and PBMCs from recruited patients and healthy controls were collected. Cells were washed twice with FACS staining buffer (PBS, 5% FBS, and 0.1% sodium azide) and stained with antibodies for 30 minutes on ice. For the detection of intracellular IL-1B, CCL3, CXCL10 and CXCR3 expression, cells were first stimulated with phorbol myristate acetate and ionomycin for 4 h with GolgiStop in complete RPMI-1640 medium in an incubator at 37 ℃ with 5% CO2. The stimulated cells were then fixed and permeabilized with a fixation/permeabilization kit (eBioscience) before intracellular staining.
Instrument	FACS Celesta.
Software	Analysis was performed in FlowJo™ v10.8 Software (BD Life Sciences).
Cell population abundance	At least, 2x10^6/ml PBMCs or SFMCs were isolated from 20 ml of the patients' whole blood, and aliquots were used

	according to the purpose of the experiments.
Gating strategy	The gating strategies are shown in Supplementary Fig. 12.<br>SFMCs: Cells (FSC/SSC) -> Singlets -> CD11b+<br>PBMCs: Cells (FSC/SSC) -> Singlets -> CD14+<br>PBMCs: Cells (FSC/SSC) -> Singlets -> CD4+

☒ Tick this box to confirm that a figure exemplifying the gating strategy is provided in the Supplementary Information.
